# Association Between EAT‐Lancet Diet Adherence and Sleep Quality in Lactating Women: A Cross‐Sectional Analysis

**DOI:** 10.1002/hsr2.73067

**Published:** 2026-08-18

**Authors:** Farzam Kamrani, Shima Jafari, Samira Karbasi, Zahra Khorasanchi

**Affiliations:** ^1^ Department of Nutrition, Faculty of Medicine Mashhad University of Medical Sciences Mashhad Iran; ^2^ Department of Clinical Pharmacy, School of Pharmacy Birjand University of Medical Sciences Birjand Iran; ^3^ Cardiovascular Diseases Research Center Birjand University of Medical Sciences Birjand Iran

**Keywords:** EAT‐Lancet diet, sleep quality, sustainable diet, women

## Abstract

**Background and Aims:**

In recent years, the sustainable EAT‐Lancet diet (ELD) has garnered attention for its health benefits, especially regarding mental health and cognitive function. However, its impact on sleep health remains underexplored. This study aims to investigate the relationship between ELD and sleep quality, focusing on lactating women, a demographic particularly susceptible to sleep disturbances.

**Methods:**

The study involved 350 lactating women aged 20–35, randomly selected from health clinics. Dietary intake was assessed using a Food Frequency Questionnaire (FFQ). ELD was evaluated using an index developed by Kesse‐Guyot, while sleep quality was measured through the self‐reported Pittsburgh Sleep Quality Index (PSQI).

**Results:**

Each one‐unit increase in ELD score was associated with 0.9% lower odds of being in a higher PSQI category in the crude model (OR = 0.991; 95% CI: 0.985–0.997; *p* = 0.003). In the adjusted model, each one‐unit increase in ELD score was associated with 0.8% lower odds of being in a higher PSQI category (OR = 0.992; 95% CI: 0.986–0.998; *p* = 0.012). Although statistically significant, the variance in PSQI explained by ELD was modest (*R* ^2 ^= 0.014), indicating that the absolute differences in sleep quality were small.

**Conclusion:**

This study is the first to show a statistically significant association between ELD adherence and sleep quality in lactating women. The effect size is modest, suggesting only slight improvements in sleep. As this is a cross‐sectional study, causality cannot be inferred, and further research is needed.

## 1 Introduction

Sleep quality includes several important aspects: sleep duration, sleep continuity, sleep depth, sleep latency, and the overall sense of restfulness and refreshment upon waking [1]. A cross‐sectional survey involving over 6000 Iranian adults revealed that 44.1% of participants experience sleep disorders [2]. Good sleep quality is crucial for overall health, particularly for lactating women, who often experience disrupted sleep patterns due to the demands of breastfeeding and caring for infants [3]. Insufficient sleep may lead to complications such as preterm childbirth [4] and is linked to chronic conditions like nonalcoholic fatty liver disease [5], inflammatory bowel disease [6], and cardiovascular disease [7].

While pharmacological interventions are available to address sleep disturbances, they may carry undesirable side effects, particularly for breastfeeding mothers [8]. Therefore, adopting proper dietary patterns is recommended as a safer and more effective approach. Plant‐based diets, particularly those rich in tryptophan, can enhance melatonin production [9]. Additionally, isoflavones found in these dietary patterns support good sleep quality [9]. Evidence suggests that a healthy diet rich in fruits, vegetables, grains, legumes, nuts, and fish is linked to a lower risk of sleep problems in mothers and infants [10].

The Food and Agriculture Organization (FAO) defines a sustainable diet as one that promotes health and well‐being while minimizing environmental impact and ensuring accessibility, affordability, safety, and cultural acceptability [11]. Following this, the EAT‐Lancet Commission proposed the EAT‐Lancet Diet (ELD), which encourages higher consumption of healthy foods like vegetables, fruits, whole grains, legumes, and nuts, while reducing intake of unhealthy options such as red meat, sugar, and refined grains [12].

Several studies have demonstrated that the ELD can improve mental health and cognitive function [13, 14, 15]. Examination of the 180,446 participants from the UK showed that lower risks of depression and anxiety by 19.4% and 18.2%, respectively [13]. In our previous study, we found that the ELD was associated with a 35% lower risk of depression in the Iranian general population [16]. The EAT‐Lancet diet, which includes legumes, nuts, seeds, and whole grains, is rich in tryptophan and limits sources of animal protein [12]. Although overall protein intake does not significantly affect sleep quality, plant‐based proteins are linked to better sleep, while certain animal proteins can have a negative impact [17]. However, the relationship between the ELD and sleep is not entirely clear‐cut.

The current study was the first to explore the relationship between adherence to the ELD and sleep quality, specifically in lactating women, highlighting the need for targeted dietary guidance in this group.

## 2 Methods

### 2.1 Study Population

This cross‐sectional research was conducted from January to February 2021 in southern Khorasan, Birjand, Iran [18, 19]. The Medical Ethics Committee at Birjand University of Medical Sciences authorized the study. Employing a stratified cluster sampling approach, 350 breastfeeding women aged between 20 and 35 years were randomly chosen from four health clinics in various parts of the city. All participants had a newborn who was between 1 and 6 months old.

The participants were asked to select their education level from the following options: no formal education, elementary school, high school graduate, bachelor's degree, master's degree, or higher. Household income level was self‐reported by participants through a subjective assessment, whereby individuals selected one of three predefined categories to describe their financial status: insufficient, sufficient, or adequate with savings. The Spielberger Anxiety Questionnaire (SAQ), which has been validated in Iran, was utilized to assess the anxiety levels of the participants [20]. This questionnaire comprises two components that independently evaluate trait and state anxiety. Each component consists of 20 questions, with each assigned a score ranging from 1 to 4. Consequently, the overall score can vary from 20 to 80 points. Body mass index (BMI) was calculated as weight in kilograms divided by the square of height in meters (kg/m^2^).

Women with chronic illnesses such as hepatic or renal failure, cardiovascular disease, autoimmune disorders, cancers, anorexia nervosa, or those who had used anti‐inflammatory, anti‐diabetic, anti‐depressant, or anti‐obesity medications in the last 6 months were excluded from the study. Additionally, participants who were underweight (BMI < 18.5 kg/m^2^) or obese (BMI ≥ 30 kg/m^2^), smoked tobacco, or took dietary supplements were not included. Prior to the commencement of any data collection, written informed consent was secured from each participant.

### 2.2 Sample Size Calculation

The required sample size was calculated using a one‐sided hypothesis test for a single mean, based on the formula: *n *= [(*Z*_1 _− *α *× SD)/*d*] [2], where SD = 5.2, *d* = 0.504, and *α* was set at 0.05 [21]. Using the corresponding *Z* value of 1.645, this yielded a minimum of 289 participants. To allow for potential exclusions and incomplete data, the enrollment was increased to 350 participants [10, 22].

### 2.3 EAT‐Lancet Diet Score Calculation

Nutritionists conducted face‐to‐face interviews to complete a 65‐item semi‐quantitative food frequency questionnaire (FFQ) to gather individuals' dietary information. Previous reports have established the reliability and validity of the FFQ within the Iranian population [23]. The average daily intake (in grams per day) for the reported items was calculated using household scales after collecting participants' information regarding the type, size, and frequency of consumption for each food item. To evaluate nutritional and energy intake, we employed Nutritionist IV software (version 7.0; N‐Squared Computing, Salem, OR), adjusted for Iranian food items.

To evaluate adherence to the ELD, we employed the EAT‐Lancet Diet Index developed by Kesse‐Guyot et al. [24]. This index assigns scores to various food groups based on their alignment with EAT‐Lancet recommendations, with higher scores reflecting a stronger adherence to the suggested dietary patterns. The index considers the intake of fruits, vegetables, whole grains, nuts, legumes, fish, and plant‐based oils while penalizing the consumption of red meat, sugar‐sweetened beverages, and starchy vegetables. Cut‐off points are detailed in other sources [24].

The equation for the ELD of an individual j can be expressed as follows:

$$\text{ELD}=\frac{100\times \left\{\sum_{\mathrm{component}\;i=1}^{14}\frac{\mathrm{ai}\times \left(\mathrm{cut}‐\mathrm{off}i-\frac{\mathrm{consumption}\;\mathrm{ij}\times 2500}{\mathrm{Energy}\;\mathrm{intake}\;j}\right)}{\mathrm{cut}–\mathrm{off}i}\right\}}{14}$$

In the above equation, ⅈ represents the 14 ELD food groups and *j* represents the individual. A value of $a_{i}=1\;$was assigned to components that should be limited, and $a_{i}=-1$ to components that should be promoted. When calculating the ELD, the result is a continuous variable (positive or negative) [25].

### 2.4 Sleep Quality Assessment

Sleep quality was evaluated using the Persian version of the Pittsburgh Sleep Quality Index (PSQI), a well‐established and validated tool designed to assess subjective sleep quality over 1 month [26]. The PSQI comprises 19 self‐rated questions organized into seven component scores: (1) subjective sleep quality, (2) sleep latency, (3) sleep duration, (4) habitual sleep efficiency, (5) sleep disturbances, (6) use of sleeping medication, and (7) daytime dysfunction. Each component score ranges from 0 to 3, with higher scores reflecting poorer sleep quality. These component scores are then summed to produce a global PSQI score that ranges from 0 to 21.

### 2.5 Statistical Analysis

Descriptive statistics were employed to summarize participants' demographic characteristics and adherence to ELD. Continuous variables were reported as means with standard deviations, whereas categorical variables were presented as frequencies and percentages. The proportional odds assumption underlying the ordinal logistic regression model was evaluated using the test of parallel lines. A non‐significant result (*p* > 0.05) was considered indicative that the proportional odds assumption was not violated. Ordinal regression was employed, and a scatter plot was utilized to represent the findings of the linear analysis graphically. The adjusted model controlled for age, BMI, anxiety, educational level, geographic area of residence (urban or rural) and household income level as potential confounders. All hypothesis tests were two‐sided with a predefined significance threshold of *p* < 0.05, and no corrections were applied for multiple comparisons.

All statistical analyses were performed using IBM SPSS Statistics for Windows, Version 26.0 (IBM Corp., Armonk, NY, released 2019).

## 3 Results

Table 1 presents descriptive information about the participants. Age did not significantly differ across the ELD tertiles (*p* > 0.05). Most participants were in sufficient conditions regarding household income levels, and this percentage was notably higher in the upper ELD tertiles compared to the first tertile. Regarding geographic location, most participants resided in the provincial center (*p* = 0.02). Furthermore, individuals in the higher ELD tertiles exhibited a greater BMI (*p* = 0.04) despite no significant differences in weight and height among the ELD tertiles (*p* > 0.05). Additionally, participants in the upper ELD tertiles displayed higher daily intakes of protein, fat, carbohydrates, and energy (*p* < 0.001). Interestingly, both anxiety scores (*p* < 0.001) and ELD scores (*p* < 0.001) increased in the higher ELD tertiles, while PSQI scores decreased (*p* = 0.005).

**Table 1:** Demographic characteristics of participants by tertiles of EAT‐Lancet diet adherence.

Variable		T1 (*N* = 125)	T2 (*N* = 105)	T3 (*N* = 120)	*p* value
Age, years		29.65 ± 6.19	29.00 ± 5.92	29.84 ± 5.66	0.29
Household income level	Insufficient	44 (35.2%)	31 (29.5%)	24 (20.0%)	0.003
	Sufficient	78 (62.4%)	73 (69.5%)	93 (77.5%)	
	Adequate with savings	3 (2.7%)	1 (1.0%)	3 (2.5%)	
Geographic area of residence	Provincial center	122 (97.6%)	97 (92.4%)	114 (95.0%)	0.02
	Urban surroundings	3 (2.4%)	4 (3.8%)	2 (1.7%)	
	Rural area	0.0%	4 (3.8%)	4 (3.3%)	
BMI, kg/m^2^		23.95 ± 4.19	26.54 ± 4.17	24.98 ± 4.44	0.04
Height, m		160.41 ± 5.75	158.91 ± 14.70	160.04 ± 11.34	0.35
Weight, kg		61.84 ± 11.76	64.28 ± 12.01	64.11 ± 12.26	0.06
Dietary protein, g/day		44.56 ± 22.20	56.10 ± 22.12	68.88 ± 32.91	< 0.001
Dietary fat, g/day		94.34 ± 49.68	129.62 ± 58.25	154.57 ± 62.53	< 0.001
Dietary carbohydrate, g/day		145.43 ± 72.53	190.76 ± 66.21	248.24 ± 121.16	< 0.001
Energy, kcal/day		1573.95 ± 751.47	2113.08 ± 781.06	2608.38 ± 1063.27	< 0.001
EAT‐Lancet diet score		5.58 ± 11.76	29.16 ± 4.15	55.13 ± 18.68	< 0.001
Anxiety score		28.92 ± 9.28	29.47 ± 10.03	31.58 ± 11.37	0.02
PSQI score		5.31 ± 2.05	5.02 ± 1.93	4.69 ± 1.86	0.005

*Note:* Values are mean ± standard deviation for continuous variables and percent for categorical variables. *p* values were derived from one‐way ANOVA for continuous variables.

Abbreviations: BMI, body mass index; PSQI, Pittsburgh sleep quality index.

**Figure 1:**
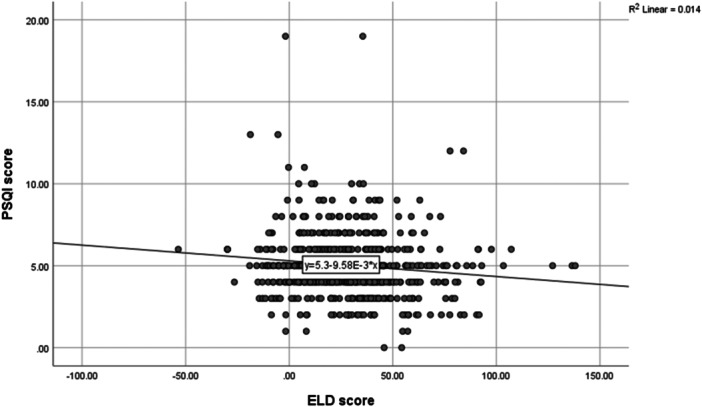
Scatter plot depicting the association between EAT‐Lancet diet (ELD) score and Pittsburgh Sleep Quality Index (PSQI) score among lactating women. This figure illustrates the linear relationship between dietary adherence to the EAT‐Lancet Diet and sleep quality, as measured by the PSQI. Each data point represents an individual participant (*n *= 350). Higher ELD scores are associated with lower PSQI scores, indicating better sleep quality. The regression line represents the fitted linear trend observed in the analysis. This scatter plot and the superimposed linear regression line are presented for visual illustration only; the primary analysis assessing the association between ELD score and sleep quality was based on ordinal logistic regression, as detailed in Table 2. ELD, EAT‐Lancet diet; PSQI, Pittsburgh Sleep Quality Index. Analysis based on linear regression.

Table 2 presents the results of the ordinal logistic regression analysis examining the association between ELD score and sleep quality. Prior to this analysis, the test of parallel lines was performed and yielded a non‐significant result (*χ* ^2 ^= 2.581, df = 4, *p* = 0.630), confirming that the proportional odds assumption was satisfied. Higher ELD scores were significantly associated with lower odds of being in a higher (worse) PSQI category in both crude and adjusted models. In the crude model, each one‐unit increase in ELD score was associated with 0.9% lower odds of being in a higher PSQI category (OR = 0.991; 95% CI: 0.985–0.997; *p* = 0.003). After adjustment for age, BMI, anxiety, educational level, geographic area of residence, and household income, this association remained statistically significant, with each one‐unit increase in ELD score corresponding to 0.8% lower odds of being in a higher PSQI category (OR = 0.992; 95% CI: 0.986–0.998; *p* = 0.012). A scatter plot illustrating the inverse association between ELD score and PSQI is presented in Figure 1. Although higher ELD adherence was significantly associated with lower odds of being in a higher PSQI category, the overall variance in PSQI explained by ELD score in a linear regression was modest (*R* ^2 ^= 0.014), indicating a small effect size.

**Table 2:** Ordinal regression results for the association between EAT‐Lancet diet score and sleep quality.

	B (95% CI)	OR (95% CI)	*p* value
Crude model	−0.009 (−0.015, −0.003)	0.991 (0.985–0.997)	0.003
Adjusted model	−0.008 (−0.014, −0.002)	0.992 (0.986–0.998)	0.012

*Note:* Adjusted model by age, BMI, anxiety, educational level, geographic area of residence (urban or rural) and household income level. The proportional odds assumption was met (test of parallel lines: *χ* ^2 ^= 2.581, df = 4, *p* = 0.630).

## 4 Discussion

The current study's findings demonstrate that each one‐unit increase in ELD score was associated with 0.8% lower odds of being in a higher (worse) PSQI category among lactating women after adjusting for potential confounders. While the association between ELD adherence and sleep quality reached statistical significance, the proportion of variance explained by the diet was small, and the absolute differences in PSQI scores across adherence categories were modest (~0.6 points on a 21‐point scale). Therefore, although the findings suggest a potential beneficial relationship, the clinical impact of ELD adherence on sleep quality is likely limited.

Research indicates that men generally sleep less than women; however, women tend to experience more disturbances during the night, particularly during early to middle adulthood, often associated with the demands of child‐rearing [27]. The lactation period can further disrupt sleep due to factors such as decreased social support, increased household responsibilities, hormonal fluctuations, and physical demands [3, 28]. A case study revealed that longer sleep durations, particularly during slow‐wave sleep, were linked to increased milk production [29]. This underscores the importance of developing interventions aimed at enhancing sleep quality for breastfeeding women.

Longer sleep duration is linked to healthier dietary patterns [30]. A systematic review study suggested that higher adherence to the Mediterranean diet is associated with better sleep quality and less insomnia [31]. Additionally, those in the highest quartile of a healthful plant‐based diet index had 0.55 times higher odds of good sleep quality, while those in the unhealthful plant‐based diet index had 2.03 times higher odds of poor sleep quality [32]. Dietary patterns can significantly affect sleep quality in various ways. Foods rich in tryptophan, such as chicken and dairy products, can enhance serotonin levels in the body. Since serotonin plays a crucial role in regulating sleep and mood, consuming these foods may improve sleep quality [33]. Additionally, hormones play a crucial role in sleep regulation. Melatonin, a hormone responsible for managing sleep‐wake cycles, can be found in foods like cherries, nuts, and oats. These foods contain either melatonin or its precursors, which can enhance the body's melatonin production and improve sleep quality [34].

ELD is considered a healthy dietary pattern that focuses more on plant‐based foods but allows moderate animal product intake. This inclusion could enhance sleep quality, as dairy products contain tryptophan, a precursor to melatonin, and are rich in micronutrients like zinc and magnesium that aid in melatonin production [35]. Additionally, ELD participants reported higher intakes of whole grains, legumes, nuts, and unsaturated fats compared to vegetarians [36], indicating their potential benefits for sleep quality. In addition to these direct mechanisms, the ELD may also influence sleep quality through several indirect pathways.

A key mechanism linking the ELD to enhanced sleep quality is its ability to reduce inflammation. Research indicates that the ELD can lower inflammation in individuals with various genetic predispositions [37]. A recent study found that compliance with the ELD was associated with decreased levels of several plasma proteins, including adrenomedullin (AM), growth differentiation factor−15 (GDF15), interleukin‐6 (IL‐6), T‐cell immunoglobulin domain and mucin domain protein (TIM), cathepsin D (CTSD), chemokine [C‐C motif] ligand 20 (CCL20), follistatin (FS), and ferric uptake regulator (FUR) [38]. Notably, elevated levels of IL‐6 have been observed in individuals with sleep disorders, such as obstructive sleep apnea [39]. Additionally, CCL20 [39] and CTSD [40] have also been linked to sleep disorders. A higher ELD score is associated with a lower asthma and airway inflammation likelihood [41]. Asthma is characterized by inflammation, and it often correlates with self‐reported insufficient sleep [42].

Another mechanism that may explain the positive association between ELD and sleep is the gut microbiome. A recent study involving German participants demonstrated a steady increase in the relative abundance of *Bifidobacterium adolescentis*, rising from 3.79% at the start to 4.9% after 12 weeks of ELD adherence [43]. Notably, clinical trials have highlighted the significant impact of *Bifidobacterium adolescentis* on improving sleep quality in adults [44]. This particular strain plays a crucial role in the production of GABA, which is essential for sleep health [45, 46].

Moreover, individuals who closely adhere to the ELD tend to report lower rates of depression and anxiety [13, 16]. Both cross‐sectional and longitudinal analyses indicate that the ELD may enhance cognitive function [15]. Evidence indicates that the highest global cognitive scores are associated with the recommended 7 h of nocturnal sleep duration [47]. Furthermore, adherence to this diet positively correlates with increased cortical thickness, indicating reduced brain atrophy in older adults [48]. Research has shown that regional cortical thinning is associated with poor self‐reported sleep quality [49]. Therefore, adherence to the ELD is associated with physiological outcomes related to brain health, including modulation of gut microbiota and reduced inflammatory mediation, which may be linked to its observed relationship with sleep quality. Interestingly, in our sample, anxiety scores were slightly higher among women with greater ELD adherence. This paradoxical finding may reflect population‐specific factors, as lactating women experience unique physiological and psychosocial stressors that could influence anxiety independently of diet. Furthermore, women with higher health awareness (and thus higher ELD adherence) may be more likely to report or monitor anxiety.

Sleep duration and quality can significantly influence dietary intake, reflecting a bidirectional relationship. For instance, a meta‐analysis has found that partial sleep restriction—defined as lasting 5.5 h or less per day—can lead to an increase in daily energy intake, including greater consumption of fats, proteins, and carbohydrates [50]. Insufficient sleep can significantly influence dietary habits in various ways. Sleep deprivation can disrupt the hormones that regulate appetite, resulting in heightened feelings of hunger and cravings for sugary, fatty, and high‐sodium foods [51]. Moreover, a lack of sleep can impair cognitive functions related to reward processing and decision‐making, making it harder to resist unhealthy food choices [52].

## 5 Strengths and Limitations

Acknowledging the critical importance of nutrition during lactation and its long‐term effects [53], this study represents the first investigation into the relationship between ELD and sleep quality among breastfeeding women. To ensure the validity of our findings, we exclusively included breastfeeding participants. We also emphasized sleep quality over sleep duration, as this approach offers a more nuanced understanding of the multifaceted nature of sleep [1].

Also, this study had certain limitations, including the potential for recall bias when utilizing the FFQ to assess dietary intake. To minimize potential confounding factors, individuals who were underweight, obese, or using dietary supplements were excluded from the study. However, these exclusions underscore the importance of conducting further research in broader populations, particularly studies that can adjust for additional confounders such as parity and physical activity, which were not available in the present dataset. Moreover, while objective methods like polysomnography (PSG) and actigraphy provide reliable data on sleep [54], self‐reported sleep quality can be susceptible to fluctuations in mood [55]. Future research should focus on incorporating these objective assessments. Furthermore, variations in scoring systems for the ELD may affect health outcomes [56], suggesting that future studies should explore alternative scoring methods related to ELD adherence. It is also important to consider that most participants in this study were urban residents. Lifestyle factors, particularly urban or rural residency, can significantly impact sleep quality [57].

## 6 Conclusion

The current study's findings indicate that adherence to the ELD, a sustainable and well‐recognized diet, is linked to improved sleep quality among lactating women. This is the first study to evaluate sleep quality as a health outcome associated with ELD adherence, particularly within the vulnerable population of lactating women, who often experience sleep deprivation and related issues. Given the cross‐sectional design of the study, causal inferences cannot be established. Therefore, further research, particularly longitudinal cohort studies and clinical trials, is needed to confirm and clarify these associations.

## Author Contributions

**Farzam Kamrani:** conceptualization, writing – original draft, formal analysis. **Shima Jafari:** data curation. **Samira Karbasi:** supervision. **Zahra Khorasanchi:** writing – review and editing, formal analysis.

## Ethics Statement

The research was approved by the Medical Ethics Committee of Birjand University of Medical Sciences (IR.BUMS.REC.1400.379) and adhered to the principles outlined in the Helsinki Declaration regarding studies involving human subjects.

## Consent

Written informed consent was obtained from all participants prior to their participation in the study.

## Conflicts of Interest

The authors declare no conflicts of interest.

## Data Availability

Data is available upon request from the corresponding author, Dr. Samira Karbasi.
